# Impact of Obesity on Outcomes of Emergency Department Visits for Cardiac Chest Pain: Insights From a Nationwide Emergency Department Study

**DOI:** 10.7759/cureus.44540

**Published:** 2023-09-01

**Authors:** Fidelis Uwumiro, Victory Okpujie, Elsie O Osiogo, Olawale Abesin, Sumayyah Abdulkabir, Aminnah Oyesomi, Grace D Ogunkoya, Abisola Bolarinwa, Chimaobi O Nwevo, Michael M Bojerenu

**Affiliations:** 1 Internal Medicine, Our Lady of Apostles Hospital, Akwanga, NGA; 2 Internal Medicine, Central Hospital Benin, Benin City, NGA; 3 Internal Medicine, Ahmadu Bello University Teaching Hospital, Zaria, NGA; 4 Internal Medicine, Royal Cornwall Hospitals NHS Trust, Cornwall, GBR; 5 Internal Medicine, National University, Khartoum, SDN; 6 Internal Medicine, Sudan International University, Khartoum, SDN; 7 Family and Community Medicine, Lagos State Primary Health Care Board, Lagos, NGA; 8 Internal Medicine, Reddington Multispecialty Hospital, Lagos, NGA; 9 Medicine and Surgery, University of Calabar Teaching Hospital, Calabar, NGA; 10 Internal Medicine, St. Barnabas Hospital (SBH) Heath System, New York, USA

**Keywords:** cardiac emergency unit, anginal chest pain, obesity paradox, cardiac sudden death, cardiac chest pain, obesity

## Abstract

Background

Obesity, a widespread national epidemic that impacts one in three U.S. adults, is closely linked with the development and exacerbation of cardiovascular disease. The objective of this study was to assess and contrast the outcomes of adults, both obese and non-obese, who present with cardiac chest pain in the emergency department (ED).

Methodology

A retrospective analysis of the 2020 Nationwide Emergency Department Sample database was conducted. Multivariate regression models were utilized to examine the association between obesity and mortality, discharge disposition, number of procedures, complications, and hospital costs.

Results

No significant difference in mortality odds was observed between obese and non-obese patients presenting with cardiac chest pain in the ED (adjusted odds ratio (aOR) = 0.92; 95% confidence interval (CI) = 0.59-1.46; p = 0.736). However, obesity was found to be associated with a decreased likelihood of being discharged home from the ED (aOR = 0.57; 95% CI = 0.52-0.63; p < 0.001), as well as an increased likelihood of hospital admission from the ED (aOR = 1.66; 95% CI = 1.53-1.81; p < 0.001). Obesity also correlated with higher odds of non-home discharge (aOR = 1.74; 95% CI = 1.54-1.97; p < 0.001), elevated mean total hospital costs (mean = $13,345 vs. $9,952; mean increase = $3,360; 95% CI = $2,816-$3,904; p < 0.001), and increased risks of cardiac arrests (aOR = 1.52; 95% CI = 1.05-1.88; p < 0.001) and acute respiratory failures (aOR = 1.43; 95% CI = 1.25-1.96; p < 0.001). Obese patients with cardiac pain underwent more procedures on average than non-obese patients (19 vs. 15; aOR = 3.57; 95% CI = 3.04-4.11; p < 0.001).

Conclusions

Obesity is associated with higher odds of hospital admission from the ED, non-home discharges, higher total hospital costs, and a greater number of procedures.

## Introduction

The age-adjusted prevalence of obesity among U.S. adults in the 2017-2018 period was 42.4%, with no notable variations between genders or age groups [1]. This indicates that nearly half of the country’s adult population has a body mass index (BMI) of 30 kg/m^2^ or higher, which classifies them as obese. According to reports, obesity-related deaths range from 262,541 to 383,410 per year, with a mean estimate of 324,940 [2]. Obesity has been identified as a contributing factor or a complicating condition in various diseases across multiple systems, including heart disease, stroke, type 2 diabetes, and some cancers [3,4]. Moreover, obesity is associated with several risk factors that can increase the chances of developing cardiac pain, such as hypertension, hypercholesterolemia, insulin resistance, chronic low-grade inflammation, and obstructive sleep apnea [5,6].

Considering the high prevalence of obesity along with its associated risks, and recognizing it as a modifiable risk factor, gaining insights into its influence on patients experiencing cardiac pain becomes invaluable. Such understanding aids in developing interventions that improve patient outcomes. This study aimed to assess and contrast outcomes between obese and non-obese patients who arrive at the emergency department (ED) with cardiac chest pain. Specifically, our study had the following objectives: first, to estimate the prevalence of obesity among adult patients presenting to the ED with cardiac chest pain; second, to analyze how obesity impacts the likelihood of mortality; and third, to ascertain the potential difference in hospital admissions, length of hospital stays, frequency of procedures, inpatient complications, and hospital costs associated with obesity among patients with cardiac chest pain. We hypothesize that obesity will correlate with poorer outcomes in the context of cardiac chest pain presentations in the ED.

## Materials and methods

Data source

The Nationwide Emergency Department Sample (NEDS) is the largest publicly available all-payer database for ED visits in the United States. In its unweighted form, the NEDS includes data from 35.8 million ED visits across hospital-owned EDs in 2020. However, when appropriately weighted, the 2020 NEDS draws upon comprehensive data from multiple states, including Arkansas, Arizona, California, Colorado, Connecticut, the District of Columbia, Florida, Georgia, Iowa, Illinois, Indiana, Kansas, Kentucky, Massachusetts, Maryland, Maine, Michigan, Minnesota, Missouri, Mississippi, Montana, North Carolina, North Dakota, Nebraska, New Jersey, Nevada, New York, Ohio, Oregon, Rhode Island, South Carolina, South Dakota, Tennessee, Texas, Vermont, Wisconsin, and Wyoming. This extensive data source enables the calculation of national estimates that effectively represent approximately 143 million ED visits throughout the United States. Importantly, this accounts for about 82.8% of the total U.S. resident population and 82.2% of all U.S. ED visits. By employing a stratification approach based on vital hospital characteristics such as geographic region, trauma center designation, urban-rural location, teaching status, and hospital ownership, the NEDS has been thoughtfully designed to facilitate research across various hospital types. Additionally, it supports the investigation of relatively uncommon disorders and procedures that may require large sample sizes for meaningful analysis.

The development of the Healthcare Cost and Utilization Project (HCUP) NEDS serves a crucial purpose, allowing for the examination of ED outcome patterns. This invaluable resource aids researchers and clinicians in making informed decisions concerning this vital source of care. Its comprehensive nature and inclusion of diverse hospital settings offer a useful resource for insightful research and evidence-based decision-making [7]. The NEDS comprehensively collects a wide range of data elements. These include details on diagnoses, such as the primary diagnosis and up to 34 secondary diagnoses. The primary diagnosis variable serves to document the primary reason for the ED visit, which helps identify the study cohort. On the other hand, the secondary diagnoses variables capture all other diagnoses, including any complications that arose during the index visit. The dataset also captures information on procedures, including the type and timing of procedures, as well as the total number of procedures performed during each ED visit. Furthermore, NEDS provides insights into the discharge status from the ED, patient demographics, payment source, hospital organizational characteristics, and more. Additionally, NEDS records pertinent information regarding any subsequent inpatient admissions following the ED visit. This includes details such as the length of the hospital stay (LOS), the total number of procedures conducted, and the associated hospital costs (THC). This extensive range of data elements allows for comprehensive analysis and exploration of various factors and outcomes from the ED setting.

Ethical consideration

The utilization of the NEDS database is subject to the regulatory framework established by the U.S. Agency for Healthcare Research and Quality (AHRQ). To maintain compliance with ethical guidelines, the design of the NEDS diligently adheres to the provisions outlined in the Healthcare Insurance Portability and Accountability Act of 1996. Since 2012, the AHRQ has implemented measures to safeguard patient and hospital information by excluding 16 direct identifiers from all NEDS datasets. As a result, the NEDS is categorized as a limited data set, which exempts it from the requirement of Institutional Review Board approval. This rigorous privacy and safety framework ensures the utmost protection and confidentiality of data, upholding the highest standards in data privacy and security [8,9].

Inclusion criteria and study variables

We queried the 2020 NEDS and included all adult ED visits for cardiac chest pain. The primary diagnoses considered for inclusion were ST-elevation and non-ST-elevation myocardial infarction, unstable angina, angina pectoris with documented spasm, other forms of angina pectoris, angina pectoris unspecified, chest pain on breathing, precordial pain, and pleurodynia. These diagnoses were identified using the International Classification of Diseases, Tenth Revision, Clinical Modification/Procedure coding system (ICD-10-CM/PCS) with specific codes (I21, I20.0, I20.1, I20.8, I20.9, R07.1, R07.2, and R07.81). The study cohort was dichotomized based on the presence or absence of a secondary diagnosis of obesity (ICD-10 code E66), with non-obese patients serving as controls for the study.

**Figure 1:**
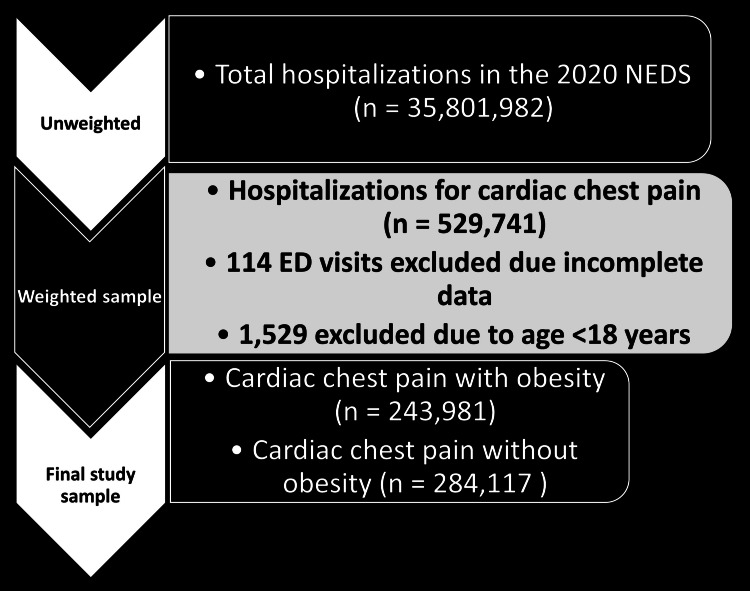
Study selection. NEDS: Nationwide Emergency Department Sample; ED: emergency department

In addition to cardiac chest pain and obesity diagnoses, our study incorporated various biodemographic variables of the patients. These variables included age, gender, race, and median annual income quartiles in the patients’ ZIP codes. Additionally, we utilized hospital-level characteristics, such as hospital region, location, bed size, and teaching status, which were already available within the NEDS database. To account for the burden of chronic medical conditions, we employed the Charlson Comorbidity Index (CCI). Specifically, we utilized the combined CCI categorized into four groups reflecting escalating mortality risk. A CCI score exceeding 3 corresponds to an approximate 25% 10-year mortality rate, while scores of 2 or 1 correspond to 10% and 4% 10-year mortality rates, respectively. We excluded patients under the age of 18 and admissions with incomplete or missing data from our study (Figure 1).

Outcome measures

The primary objective of our study was to determine the probability of mortality among patients with cardiac chest pain, assessing the impact of obesity. Additionally, we examined several secondary outcomes, including the duration of both ED and subsequent inpatient stays, discharge destinations, associated costs, the number of procedures performed, as well as the odds of experiencing cardiac arrest or respiratory failure. In the NEDS database, mortality is recorded using a variable labeled as “DIED_VISIT.” Within this variable, a value of 0 indicates survival during the initial visit, while a value of 1 signifies mortality within the ED, and a value of 2 indicates death after being admitted to inpatient care.

To further explore secondary outcomes, we employed relevant ICD-10 CM/PCS codes obtained from the secondary diagnoses variables. These codes included I46.2, I46.9, R09.2, and J96.9, which were used to identify the likelihood of cardiac arrests and respiratory failure. We also assessed additional secondary outcomes, such as the average LOS following inpatient admission, the mean THC (comprising both ED and inpatient services), and the odds of multiple procedures (defined as the presence of 10 or more hospital procedures performed on a single patient either during the initial ED visit or subsequent inpatient admission). The LOS, THCs, and number of procedure variables are numerical indicators that are already present within the NEDS database. The procedures performed in each ED visit or any subsequent inpatient admission are recorded within 15 procedure variables (denoted “I10_PR_IP1 - I10_PRI_IP15”) in the NEDS database allowing for easy estimation of total procedures performed.

Statistical analyses

We conducted the statistical analyses using Stata, version 17.0MP (StataCorp LLC, College Station, TX, USA). Unadjusted odds ratios (ORs) were computed for both primary and secondary outcomes through univariate logistic regression analyses. For subsequent multivariate logistic regression modeling, variables with p-values <0.1 were selected. This criterion, especially considering the extensive sample size, aimed to limit the inclusion of marginally relevant factors that do not significantly impact the outcomes of interest. Pearson’s chi-square tests were utilized to compare proportions among nominal variables, while Student’s t-test was employed for continuous variables. Throughout all multivariate analyses, statistical significance was determined at p-values <0.05. Categorical variables were presented as proportions, while continuous variables were reported as mean values with their standard deviations. The results of the regression analyses were reported as adjusted odds ratios (aORs) or β coefficients, each with their corresponding 95% confidence intervals (CIs). To account for confounders in the secondary outcomes, multivariate logistic and linear regression models were utilized, incorporating patient and hospital variables as well as comorbidities.

Data availability statement

Publicly available NEDS datasets were used in this study. These can be assessed through the AHRQ’s online HCUP central distributor at https://www.ahrq.gov/data/hcup/index.html.

## Results

Baseline biodemographic and hospital characteristics

Our study included 528,098 adult ED visits for cardiac chest pain. About 114 visits with incomplete or missing data were excluded from our study. Among the ED visits analyzed, 243,981 (46.2%) had a secondary diagnosis of obesity, while 284,117 (53.8%) served as controls. Obese patients with cardiac pain tended to be slightly older (53.7 years vs. 50.5 years, p < 0.001) and female (59.4% vs. 40.6%, p < 0.001) compared to non-obese patients. Obese patients admitted with cardiac chest pain had a higher comorbidity burden compared to non-obese patients. This included diabetes mellitus (27.4% vs. 12.9%, p < 0.001), COVID-19 (13.4% vs. 6.5%), peripheral vascular disease (4.4% vs. 2.5%, p < 0.001), coronary artery disease (10.4% vs. 6.0%, p < 0.001), hypertension (23.5% vs. 9.5%, p < 0.001), congestive heart failure (11.8% vs. 5.7%, p < 0.001), chronic kidney disease (8.4% vs. 4.2%, p < 0.001), and chronic obstructive pulmonary disease (20.9% vs. 12.4%, p < 0.001). Conversely, patients with dementia were more commonly found in the non-obese subgroup (1.1% vs. 0.8%, p < 0.001) compared to the obese subgroup (Table 1).

**Table 1:** Sociodemographic characteristics of the study population. USD: U.S. dollar; CCI: Charlson Comorbidity Index; COPD: chronic obstructive pulmonary disease

Variable	Cardiac pain with obesity (n = 243,981), n (%) unless otherwise specified	Cardiac pain without obesity (n = 284,117), n (%) unless otherwise specified	P-value
Women	144,680 (59.3)	156,866 (55.2)	<0.001
Mean age, years	53.7	50.5	<0.001
Age categories, years			<0.001
18–39	39,281 (16.1)	152,319 (53.6)	
40–64	148,096 (60.7)	136,121 (47.9)	
≥65	55,140 (22.6)	65,929 (23.2)	
Weekend admission	57,336 (23.5)	68,771 (24.2)	0.002
CCI score			<0.001
0	39,523 (16.2)	67,634 (23.8)	
1	20,738 (8.5)	17,335 (6.1)	
2	9,027 (3.7)	6,536 (2.3)	
≥3	174,690 (71.6)	192,672 (67.8)	
Insurance type			<0.001
Medicaid	77,342 (31.7)	80,138 (28.2)	
Medicare	47,576 (19.5)	61,951 (21.8)	
Private	91,249 (37.4)	98,609 (34.7)	
Uninsured	27,814 (11.4)	43,479 (15.3)	
Median annual income in patient’s zip code (USD^*^)			<0.001
1–43,999	92,469 (37.9)	99,462 (35.8)	
44,000–55,999	69,779 (28.6)	78,149 (27.5)	
56,000–73,999	49,538 (20.3)	57,404 (20.2)	
≥74,000	32, 449 (13.3)	46,605 (16.4)	
Comorbidities			
Old myocardial infarction	25,374 (10.4)	17,050 (6.0)	<0.001
Hypertension	57,336 (23.5)	26,997 (9.5)	<0.001
Congestive heart failure	28,790 (11.8)	16,198 (5.7)	<0.001
Peripheral vascular disease	10,735 (4.4)	7,104 (2.5)	<0.001
Cerebrovascular disease	4,148 (1.7)	2,273 (0.8)	<0.001
Dementia	1,952 (0.8)	3,126 (1.1)	<0.001
COPD	50,992 (20.9)	35,238 (12.4)	<0.001
Rheumatoid disease	5,612 (2.3)	3,694 (1.3)	<0.001
Peptic ulcer disease	732 (0.3)	568 (0.2)	<0.001
Gastroesophageal reflux disease	62,459 (25.6)	15,914 (5.6)	<0.001
Liver disease (mild)	6,344 (2.6)	2,842 (1.0)	<0.001
Liver disease (moderate to severe)	220 (0.09)	142 (0.05)	<0.001
Uncomplicated diabetes	66,850 (27.4)	36,659 (12.9)	<0.001
Complicated diabetes	22,690 (9.3)	8,809 (3.1)	<0.001
Hemiplegia or paraplegia	488 (0.2)	284 (0.1)	<0.001
Chronic renal disease	20,494 (8.4)	11,935 (4.2)	<0.001
Cancer	2,439 (1.0)	2,273 (0.8)	<0.001
Metastatic cancer	488 (0.2)	568 (0.2)	0.181
AIDS	244 (0.1)	568 (0.2)	0.156
Leukemia	488 (0.2)	284 (0.1)	0.954
Lymphoma	24 (0.01)	568 (0.2)	<0.001
COVID-19	32,693 (13.4)	18,472 (6.5)	0.03
Hospital characteristics			
Hospital region			<0.001
Northeast	27,570 (11.3)	49,163 (17.3)	
Midwest	64,655 (26.5)	65,929 (23.2)	
South	111,987 (45.9)	117,081 (41.2)	
West	39,769 (16.3)	52,004 (18.3)	
Urban location	212,263 (87.0)	235,583 (82.9)	0.156
Teaching hospital	192,257 (78.8)	218,532 (76.9)	<0.001

Regarding geographic location and hospital type, we found that cardiac chest pain admissions with obesity were most frequently recorded in hospitals in the South (37.9%), followed by the Midwest (28.8%), Northeast (19.4%), and Western (13.9%) regions. The majority of these hospitalizations (77.7%) occurred in teaching hospitals. Similarly, for non-obese cardiac pain admissions, the highest proportion was observed in the South (37.9%), followed by the Midwest (23.9%), Northeast (21.7%), and Western (16.5%) hospital regions (Table 1).

Primary outcome: mortality

There was no statistically significant difference in the odds of mortality between admissions for cardiac chest pain with or without obesity (aOR = 0.92; 95% CI = 0.59-1.46; p = 0.736) (Table 2).

**Table 2:** Multivariable regression analyses of outcomes of cardiac pain hospitalizations with and without obesity. *: Statistically significant at p < 0.05. ^a^: aORs for prolonged hospital length of stay (defined as the length of stay in the top decile for all adult ED visits for cardiac chest pain). ^b^: Adjusted mean increase. ^c^: All ED visits resulting in admission or transfers other than routine home discharge. ^d^: Inpatient discharges or transfers other than routine home discharge. aOR: adjusted odds ratio; CI: confidence interval; ED: emergency department; US$: U.S. dollar

Outcome	Cardiac pain with obesity, (%)	Cardiac pain without obesity, (%)	aOR (95% CI)	P-value^*^
Primary outcome				
Mortality	222 (0.091)	269 (0.095)	0.92 (0.59–1.46)	0.736
Secondary outcomes				
Mean length of inpatient stay, days	2.9	1.2	1.50^a ^(1.42–1.63)	<0.001
Mean total hospital charge (US$)	13,345	9,952	US$3,360^b^ ($2,816–$3,904)	<0.001
ED non-release^c^	35,377 (14.5)	27,849 (9.8)	1.66 (1.53–1.81)	<0.001
Inpatient non-release^d^	4,392 (1.8)	3,410 (1.2)	1.74 (1.54–1.97)	<0.001
Cardiac arrest	1,952 (0.8)	57 (0.02)	1.52 (1.05–1.88)	0.021
Acute respiratory failure	2,196 (0.9)	1,705 (0.6)	1.43 (1.45–1.96)	0.025
≥10 procedures	41,477 (17)	22,734 (8)	3.57 (3.04–4.11)	<0.001

Secondary outcomes

Obese patients had longer LOS compared to the non-obese patients (mean LOS of 2.9 days vs. 1.2 days). After adjustments, obese patients were more likely to be in the top decile for hospital LOS compared to the non-obese patients (aOR = 1.50; 95% CI = 1.42-1.63; p < 0.001) and less likely to be discharged home (adjusted odds of inpatient admission from the ED = 1.66; 95% CI = 1.53-1.81; p < 0.001). Furthermore, among patients admitted from the ED to inpatient care, obese patients had greater odds of discharge to skilled nursing homes and other acute care hospitals (aOR = 1.74; 95% CI = 1.54-1.97; p < 0.001) (Table 2) and higher mean THCs ($13,345 vs. $9,952; mean increase = $3,360; 95% CI = $2,816-$3,904; p < 0.001) compared to non-obese patients. Private insurance was the highest primary payer, covering 34.7% of the cases, followed by Medicaid (28.2%) and Medicare (21.7%). A smaller proportion of patients (15.4%) were uninsured. Additionally, a large proportion of cardiac pain hospitalizations with obesity were recorded among individuals belonging to the $1-$43,999 (low) median annual income group (37.9%), followed by the $56,000-$73,999 (28.6%), $44,000-$55,999 (20.3%) and ≥$74,000 (13.3%) median annual income groups (p < 0.001).

The risk of cardiac arrests (aOR = 1.52; 95% CI = 1.05-1.88; p = 0.021) and acute respiratory failures (aOR = 1.43; 95% CI = 1.45-1.96; p = 0.025) was significantly higher for obese patients who were admitted with cardiac chest pain compared to non-obese patients. Obese patients had a higher mean number of procedures performed during the index hospitalization in comparison to non-obese patients (17 vs. 8, aOR = 3.57; 95% CI = 3.04-4.11; p < 0.001).

## Discussion

Our study investigated the differences in clinical outcomes and resource utilization between obese and non-obese patients presenting with cardiac pain to the emergency department. The results showed that there was no statistically significant difference in the odds of mortality between obese and non-obese patients admitted to the ED for cardiac pain. This is consistent with previous studies that have suggested that despite the link between obesity and the onset of cardiovascular disease, obesity may not be an independent predictor of mortality in patients with cardiovascular diseases (the obesity paradox) [10-12]. However, our findings showed that obese patients were less likely to be discharged home from the ED and had higher odds of inpatient admission. This may be due to the increased comorbidities associated with obesity, such as diabetes and hypertension, which may require more extensive evaluation and treatment [13,14]. Additionally, we found that among patients admitted to in-hospital care, obesity was associated with a higher likelihood of non-home discharge and increased mean total inpatient charges compared to non-obese patients. This may be explained by the increased healthcare resource utilization required to manage the complex medical needs of obese patients [15,16].

Another important finding of our study is the significantly augmented risk of cardiac arrests and acute respiratory failures associated with obesity. This observation implies that individuals with obesity may encounter more severe illness, necessitating increased vigilance and enhanced management within the ED to avert adverse outcomes. Additionally, our analysis revealed that patients with obesity underwent a greater number of procedures during their initial hospital stay compared to non-obese patients. This trend might mirror the requirement for more interventions within this group, possibly stemming from the augmented burden of comorbidities found among individuals with obesity. The longer mean LOS for obese patients observed in our study is an unclear finding that requires further investigation. It may reflect differences in the severity or complexity of the underlying medical conditions or differences in the response to treatment between the two groups.

Our findings have important implications for the management of patients with obesity in the ED and highlight the need for further research to better understand the impact of obesity on clinical outcomes in this population. Additionally, strategies aimed at reducing healthcare resource utilization, reducing hospital stay, and improving outcomes in obese patients may be beneficial in managing this population. Studies have reported that acute care settings are inadequately equipped to provide optimal care for patients classified as obese. These settings lack the dedicated equipment required to cater to the special needs of these patients. In fact, most facilities have been retrofitted to make do, rather than being specifically designed for this purpose, resulting in inadequate care provision [17,18].

For obese patients who present with cardiac chest pain, several specific areas of focus may be necessary. Obese patients presenting with cardiac pain require tailored interventions to minimize the impact of obesity on cardiovascular disease. These interventions include weight management programs, nutritional counseling, exercise recommendations, medication optimization for obesity, behavioral modifications, psychosocial support, bariatric surgery evaluation, care coordination, patient education, and regular monitoring. Specialized weight management programs may provide comprehensive support through dietary counseling, physical activity recommendations, and behavioral assistance to obese patients. Nutritional counseling is useful to educate patients about healthy eating habits and portion control. Exercise recommendations focus on low-impact activities and gradual increases in intensity. It may be useful to consider low-impact activities that minimize stress on joints, such as swimming, walking, or cycling and gradually increasing exercise intensity and duration while monitoring the patient’s cardiovascular response. Optimizing medication management for obese patients with cardiac pain, taking into account their body weight, potential interactions with obesity-related medications, and specific cardiovascular conditions, may be helpful. In addition, implementing behavioral modification techniques will help obese patients adopt healthier lifestyle behaviors. This may involve setting realistic goals, addressing emotional eating, stress management, and promoting adherence to medication and lifestyle changes. Psychosocial support, including counseling and support groups, also helps address psychological factors in obesity [19,20].

The stigma surrounding obesity is particularly pronounced in healthcare. Weight stigma, characterized by negative stereotypes, biases, and discrimination against individuals based on their weight, exerts detrimental effects on patient care and outcomes. Unfortunately, this pervasive stigma contributes to various consequences, including the avoidance of healthcare services, delayed diagnosis, and postponed presentations of potentially life-threatening conditions [21]. When examining weight stigma through the lens of healthcare, it becomes evident why a higher proportion of obese patients tend to present to the ED with cardiac chest pain compared to the prevalence in the general population. The discriminatory attitudes and judgments encountered by individuals with obesity may discourage them from seeking timely medical attention. Fear of being stigmatized or experiencing negative interactions with healthcare providers may lead to the avoidance of healthcare altogether. As a result, essential interventions or treatments are delayed, potentially postponing accurate diagnoses and impeding effective healthcare delivery, thereby worsening the severity of their conditions. Stereotypes surrounding obesity may lead healthcare providers to attribute symptoms solely to weight-related factors, overlooking underlying medical issues. These delayed or missed diagnoses can have severe consequences, especially when dealing with life-threatening conditions that require immediate medical attention. The repercussions of such delayed presentations can be devastating, with potentially negative impacts on patient outcomes and quality of life [22].

The data from the index study strongly suggest that the association between obesity and increased presentations of cardiac chest pain in the ED may be influenced by weight stigma. This finding underscores the urgent need to address weight stigma in healthcare settings and ensure equitable access to timely and appropriate care for obese individuals. To mitigate the negative consequences of weight stigma in healthcare, several strategies can be implemented. First, healthcare providers require additional education and training to develop awareness and sensitivity toward weight-related issues. This includes encouraging a non-judgmental and supportive environment that promotes open communication and patient-centered care. Healthcare systems should implement policies and guidelines that address weight bias, promote inclusive practices, and ensure equitable access to healthcare services for individuals with obesity. Efforts to combat weight stigma should extend beyond healthcare providers and systems. Raising awareness and promoting public discourse about the harmful impact of weight stigma is crucial for effecting societal change. It is essential to challenge societal norms and attitudes that perpetuate weight bias, promoting a culture of acceptance and respect for all individuals, regardless of their weight or size [23]. Overall, establishing a regular follow-up schedule to monitor patients’ progress, provide ongoing support, and make necessary adjustments to patient treatment plans combined with regular monitoring of weight, blood pressure, lipid levels, and other relevant markers within a supportive environment devoid of stigmatization can help track improvements and guide interventions. As a last resort, bariatric surgery evaluation may be considered for eligible patients [24].

Limitations

This study had some limitations that should be acknowledged. First, NEDS data only reflects the year of the claim and may not reflect the long-term health outcomes of this patient population. It is also worth mentioning that the prevalence of obesity in this study may have been slightly underestimated as some U.S. hospitals do not contribute data to the NEDS. Due to the limitations of ICD-10 codes, the proportions of patients with pulmonary embolisms and their various causes were not estimated. Nevertheless, we employed the largest available all-payer ED database and robust statistical methodologies to gain useful insights into the experiences of this important patient population.

## Conclusions

While there was no significant difference in mortality rates between obese and non-obese patients, obesity remained linked to higher resource utilization and poorer outcomes among those who present at the ED due to cardiac chest pain. Additional research is needed to refine and extend these findings, as well as to establish evidence-based interventions that effectively tackle the intricacies of managing cardiac chest pain in individuals with obesity. Through an ongoing commitment to enhancing healthcare delivery and fostering a patient-centric approach, greater strides can be taken to alleviate disparities, reduce stigma, and enhance outcomes for individuals with cardiac conditions linked to obesity, both within the ED and beyond.
